# Replacement of Dislodged Gastrostomy Tubes After Stoma Dilation in the Pediatric Emergency Department

**DOI:** 10.5811/westjem.2017.3.31796

**Published:** 2017-04-19

**Authors:** Shiloni Bhambani, Tammy H. Phan, Lance Brown, Andrea W. Thorp

**Affiliations:** * Kaiser Permanente Medical Center, Department of Pediatrics, Fremont, California; † Kaiser Permanente Medical Center, Department of Emergency Medicine, Fontana, California; ‡ Loma Linda University Medical Center, Loma Linda, California; § Loma Linda University Medical Center and Children’s Hospital, Department of Emergency Medicine and Pediatrics, Loma Linda, California

## Abstract

**Introduction:**

A dislodged gastrostomy tube (GT) is a common complaint that requires evaluation in the pediatric emergency department (ED) and, on occasion, will require stoma dilation to successfully replace the GT. The objective of this study was to describe the frequency that stoma dilation is required, the success rate of replacement, complications encountered, and the techniques used to confirm placement of the GT after dilation.

**Methods:**

We conducted a retrospective medical record review of children 0–18 years who presented to the pediatric ED from February 2013 through February 2015 with a dislodged GT that required stoma dilation by pediatric emergency physicians with serially increasing Foley catheter sizes prior to successful placement of the GT.

**Results:**

We reviewed a total of 302 encounters in 215 patients, with 97 (32%) of the encounters requiring stoma dilation prior to replacing a GT. The median amount of dilation was 2 French between the initial Foley catheter size and the final GT size. There was a single complication of a mal-positioned balloon that was identified at the index visit. No delayed complications were encountered. We performed confirmation of placement in all patients. The two most common forms of confirmation were aspiration of gastric contents (56/97 [58%]) followed by contrast radiograph in 39 (40%).

**Conclusion:**

The practice of serial dilation of a gastrostomy stoma site to allow successful replacement of a gastrostomy tube in pediatric patients who present to the ED with a dislodged gastrostomy tube is generally successful and without increased complication. All patients received at least one form of confirmation for appropriate GT placement with the most common being aspiration of gastric contents.

## INTRODUCTION

The most common minor complication for patients with a gastrostomy tube (GT) is dislodgement.^1^ It is estimated that 2% will become displaced within the first 10 months after placement and 37% dislodged within five years.^2^,^3^ Of the patients who present to a pediatric emergency department (ED), 62% of GT-related complaints are dislodged tubes and most of these require replacement prior to discharge.^1^ Parents who feel uncomfortable with the replacement of the GT at home or those who had a difficult time replacing the GT themselves bring their child to the ED for evaluation.^4^ It is well described that replacement, with or without confirmation imaging, is a procedure commonly performed successfully in the ED.^1^,^4^,^5^ There is an estimated complication rate between 1–4% with GT replacements performed in the ED.^4^,^6^ These complications include over-filled or mal-positioned balloon, gastric outlet obstruction, and peritonitis.^1^,^4^ However, if the stoma site has partially closed, the GT may be difficult to replace. One of the techniques that allows for successful replacement is serial dilation of the stoma with progressively larger Foley catheters prior to replacing the GT.^5^ It has been estimated that nearly 33% of pediatric stoma sites require dilation prior to replacing the GT,^1^ but no study describes the success rate of GT placement after stoma dilation or the complications that occur after dilation. Of the replaced GTs in pediatric patients in the ED, it is estimated that 35% of cases will have imaging to confirm GT placement.^4^ As a secondary outcome, we describe the types of confirmation obtained and the percentage of patients who received confirmation when stoma dilation had been performed.

## METHODS

We conducted a retrospective electronic chart review of all children ages 0–18 years who presented to our pediatric ED from February 2013–February 2015 for replacement of a dislodged GT that required serial dilation for successful replacement by a pediatric emergency physician (EP). The pediatric ED is a Level I trauma center with approximately 25,000 visits annually. Our hospital switched to a new electronic medical record (EPIC) mid-February; therefore, this start date was used rather than a typical calendar year. Charts were identified using a search of chief complaints that included feeding tube or gastrostomy tube, the Current Procedural Terminology (CPT) code 43760 for a change of gastrostomy tube, and diagnosis code V55.1 attention to gastrostomy. We excluded children if the stoma site did not require dilation, when the GT was replaced by another feeding tube besides a GT (nasogastric tubes, gastrojejunostomy tubes, or Foley catheters that were left in place at the time of discharge from the ED), and when the GT was replaced by either pediatric surgery, parents, or the pediatric gastroenterology service prior to any attempts being made by pediatric EPs. For those patients with multiple visits for recurrent dislodgement of the GT, we included all visits unless the repeat visit was deemed to be a result of a complication from the index visit.

Using a standard data collection form, a single researcher (SB) extracted all data from the electronic medical record. Data collected included demographic data, age of stoma tract, length of time the GT had been dislodged prior to arrival, initial Foley catheter size, final GT size, immediate complications, and techniques used to confirm GT placement. Delayed complications were investigated by reviewing each chart for any return ED visits with a diagnosis that included abdominal pain, abdominal distention, vomiting, or intolerance of GT feedings after the index visit. We used descriptive statistics within Excel to analyze the data. Our institutional review board approved this study.

**Population Health Research Capsule**

What do we already know about this issue?

Dislodged gastrostomy tubes commonly present to the pediatric emergency department and some will require serial dilation to replace the tube successfully.

What was the research question?

What is the success rate of gastrostomy tube placement after stoma dilation and what, if any, complications occur after dilation?

What was the major finding of the study?

Serial dilation of a gastrostomy stoma site to allow successful replacement of a gastrostomy tube is generally successful and without increased complication.

How does this improve population health?

Performing serial dilation by emergency physicians with successful replacement of the gastrostomy tube could potentially decrease ED length of stay and surgical consultations.

## RESULTS

We reviewed a total of 215 patient charts with 302 encounters for GT-related complaints. Of those, 261 (86%) had a dislodged feeding tube and 97 encounters (32%) required stoma dilation prior to replacement of the GT (Figure). The most commonly placed initial Foley catheter size was 10 French with a median increase in dilation by 2 French between the initial Foley size and the final GT size (Table 1). Thirteen patients (13%) required dilation 1 Foley size (2 French) larger than the target GT size to allow successful GT placement.

Of the 97 encounters, 96 (99%) of the GTs were successfully replaced by a pediatric EP after serial stoma dilation and on the first replacement attempt. One patient was found to have a mal-positioned and over-filled balloon on confirmation contrast imaging that required a second replacement attempt by a pediatric EP. The balloon was repositioned and filled with 3 ml instead of the previous 4 ml of normal saline. Gastric contents were aspirated after adjusting the GT and the patient was discharged home during that index visit. We considered this case an immediate complication giving a complication rate of 1/97 patients (1%, 95%CI [0.03–5.6]). In addition, there were no failed replacement attempts by pediatric EPs that required consultation with pediatric surgery or pediatric gastroenterology during the index visit.

There were no delayed complications or return ED visits with symptoms that could be reasonably associated with the stoma dilation. The only return visit was a patient who returned four days later with abdominal pain and vomiting. This patient was admitted for chronic ileus related to underlying anal stenosis and was admitted to pediatric surgery for anal dilation. Upon review of the hospital chart, the dilation of the stoma and replacement of the GT during the index ED visit was not thought to be a contributing factor to the symptoms at the return visit.

The methods used to confirm successful GT replacement were described as a secondary comparative outcome. All patients had at least one form of confirmation mentioned in the chart. The two most common forms of confirmation were aspiration of gastric contents in 56 (58%) of the patients and contrast radiograph in 39 (40%) of the patients (Table 2). Of the 97 encounters, 30 (31%) had two forms of confirmation, nine (9%) had three forms of confirmation and one (1%) had four forms of confirmation. For the one patient who had the immediate complication, two forms of confirmation were used during the index visit.

## DISCUSSION

The percentage of dislodged GTs that presented to our pediatric ED was slightly higher compared to previous publications (86% v 62%).^1^ However, approximately one third of our patients required dilation of the stoma to successfully replace the GT, and this seems to be similar to previous pediatric reports.^1^ Complication rates are estimated to be around 1–4 % for all GTs placed in the ED, and stoma dilation in our pediatric study did not result in a higher rate of complication.^4^,^6^ The one immediate complication in our study population is a known complication for any inserted GT and cannot be solely attributed to the stoma dilation itself. Furthermore, none of the patients had return ED visits with delayed complications that could be attributed to the dilation of the stoma tract. Given the high frequency of dislodged GTs and the fact that stoma dilation can be performed without increased complication rate, it seems reasonable that stoma dilation to successfully replace a GT in a pediatric patient should be within the scope of emergency medicine practice.

Previous reports mention that approximately one third of the GTs replaced in the ED receive confirmation of placement with contrast radiographs.^4^ We had a slightly higher rate of confirmation radiographs in our study after stoma dilation. A majority of our patients received a single form of placement confirmation and a majority of those had aspiration of gastric contents as their only form of confirmation. Making a recommendation for confirmatory imaging when the stoma is dilated is beyond the scope of this article; however, the rate of complications after stoma dilation seems to be similar to previously reported rates of complication for any GT replacement. Therefore, it seems reasonable that performing dilation of the stoma should not change a physician’s practice of confirming GT placement. In other words, it is reasonable for a physician to continue performing the confirmation technique of their choice regardless of the stoma being dilated prior to GT placement.

Stoma sites can start to tighten within hours of the GT falling out, and the technique of using a catheter to keep the stoma open followed by serial stoma dilation has been previously described by Willworth.^5^ When the GT cannot be replaced because the stoma site is closing, a Foley catheter should be placed in the stoma as soon as possible to keep the stoma from tightening further or completely closing. If the largest catheter that can be placed without traumatizing the stoma site is smaller than the target GT size, dilation of the stoma tract is required. Serial dilation involves removing the initial Foley catheter followed by immediate replacement with the next largest Foley catheter (2 French increase) until the target GT-size Foley catheter is reached. A small portion of our study population required dilation 1 Foley larger than the target GT. Although this has not been studied, it seems reasonable to conclude that the balloon adds a small amount of diameter beyond the designated size of the catheter. Therefore, placing a Foley catheter that is 2 French larger beyond the target GT size will dilate the stoma to a size that could account for the balloon width. After placement of each Foley catheter, clamping or kinking the catheter is necessary to keep the gastric contents from leaking. There is no specific or accepted time that each Foley needs to stay in the stoma prior to changing to the next larger Foley. None of the charts in our study mentioned how long each Foley stayed in the stoma site. Anecdotally, it was noticed that the length of time each Foley stays in the stoma is directly related to the volume of patients in the ED at the time.

## LIMITATIONS

This study was a retrospective chart review, a methodology with inherent risk of missing patients who could have been included or missing data that could have altered our conclusions. However, the impact of this missed data is estimated to be minimal. We encountered a single complication, and stating a complication rate based on a small number of adverse outcomes or making conclusions about causation between stoma dilation and the single complication encountered could be seen as problematic. When evaluating delayed complications, only return ED visits to our institution were reviewed. It is possible that patients could have presented to other hospitals with abdominal complications that could be attributed to stoma dilation; however, it is customary for complex pediatric patients to be transferred back to our tertiary care ED after presenting to a regional hospital. This is a single-center study at a tertiary pediatric ED and the results may be difficult to generalize to other institutions or clinicians who may feel uncomfortable with this procedure in a pediatric patient. Each pediatric EP who treated the patients in our study chose the technique of GT placement confirmation with which they were comfortable. Whether more or fewer confirmatory studies should be done after stoma dilation would require further study.

## CONCLUSION

The practice of serial dilation of a gastrostomy tube stoma site to allow successful replacement of a gastrostomy tube in pediatric patients who present to the ED with a dislodged gastrostomy tube is generally successful and without increased complication. All patients received at least one form of confirmation for appropriate GT placement with the most common being aspiration of gastric contents.

## Figures and Tables

**Figure:**
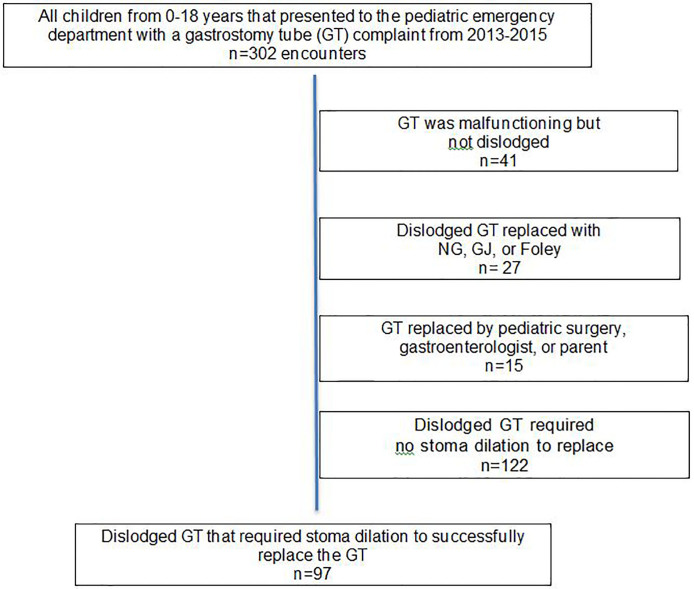
Flow diagram of pediatric patients who presented to the ED with gastrostomy tube complaint. *GT,* gastrostomy tube.

**Table 1:** Demographics and description of gastrostomy stoma dilation (n=97).

		Range of values
Median age of patient (mo)	34	2 months – 17 years
Median age of GT stoma tract (mo) *	12	2 months – 17 years
Median time GT dislodged prior to ED evaluation (hrs) *	3	1 – 22 hours
Mode initial Foley size (Fr) *	10	8–14 Fr
Mode final GT size (Fr)	12	10–18 Fr
Median increase from initial Foley to final GT size (Fr) +	2	0 – 8 **

* One patient with missing information.

+ Unable to calculate for the one patient with missing information regarding initial Foley size.

** Increase of 0 was seen in three patients with the initial Foley size and final GT size being the same, but all those patients required dilation with a Foley size 2 French larger than the final GT to successfully replace the GT.

**Table 2:** Methods of confirmation regarding placement of gastrostomy tube (n=97).*

Form of GT confirmation	No. of encounters (%)
Aspiration of gastric contents	56 (58%)
Contrast radiograph	39 (40%)
Tolerating feed	33 (34%)
Listened for air inflation	17 (18%)
Normal abdominal exam	2 (2%)

* Total number of encounters in the table will equal more than 97 since 40 of those encounters obtained two or more forms of confirmation.
